# Supplementary material for: The Influence of Gender on Long-Term Cardiovascular Outcomes in Patients Undergoing Percutaneous Coronary Intervention for Acute Myocardial Infarction and the Association with Cardiac Left Ventricular Function
Source: Diagnostics (Basel). 2025 Nov 16;15(22):2901. doi: 10.3390/diagnostics15222901 (PMC12651039; doi:10.3390/diagnostics15222901)
Supplement: Supplementary file 1 [file diagnostics-15-02901-s001.zip › diagnostics-3939173-Supplementary Materials.pdf]

diagnostics

MDPI

Supplemental file

Figure S1: Flow chart on 289 patients screened for inclusion with follow-up times for the telephone interview and to end-of follow-up

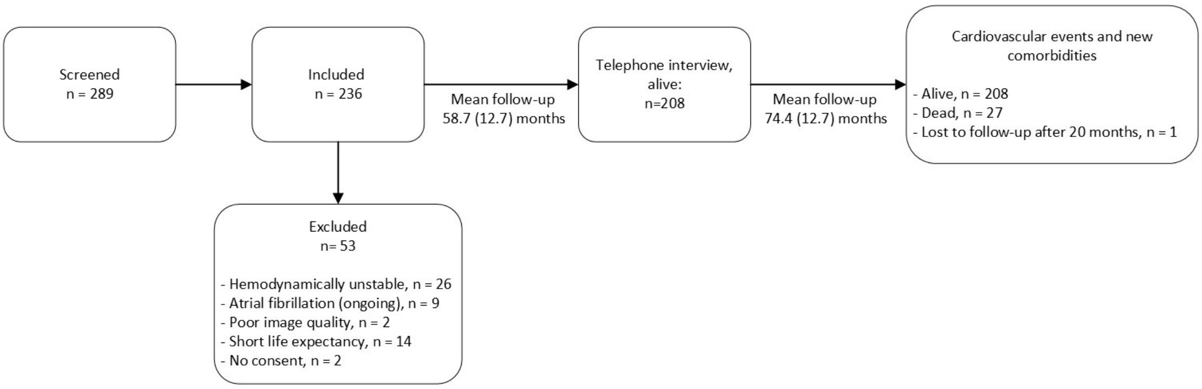

Table S1: Age and baseline echocardiographic variables among all patients subdivided into anterior wall STEMI and non-anterior wall STEMI plus NSTEMI. Data presented as mean (SD)

Variable	anterior STEMI, n=55	non-anterior STEMI + NSTEMI, n=181	p-value
Age, years	63 (11)	65 (10)	n.s.
LVEF, %	46 (8)	51 (8)	p<0.001
LVEDVI, ml/m2	87 (18)	81 (20)	n.s.
LVESVI, ml /m2	47 (15)	41 (15)	p<0.01
GLS %	-12.3 (3.0)	-14.9 (3.3)	p<0.001

Abbreviations: LVEF = left ventricular ejection fraction; LVEDVI = left ventricular end diastolic volume index; LVESVI = left ventricular send systolic volume index; GLS = global left ventricular strain (expressed as positive values)

Table S2: Pearsons correlation analysis of echocardiographic left ventricular indices. Numbers express two-tailed significance

Variable	LVEF	LVEDVI	LVESVI	GLS
LVEF		$p < 0.001$	$p < 0.001$	$p < 0.001$
LVEDVI	$p < 0.001$		$p < 0.001$	$p < 0.001$
LVESVI	$p < 0.001$	$p < 0.001$		$p < 0.001$
GLS	$p < 0.001$	$p < 0.001$	$p < 0.001$	

Abbreviations: LVEF = left ventricular ejection fraction; LVEDVI = left ventricular end diastolic volume index; LVESVI = left ventricular send systolic volume index; GLS = global left ventricular strain (expressed as positive values)

 https://doi.org/10.3390/diagnostics15222901
